# Editorial: Systemic implications of Alzheimer's disease

**DOI:** 10.3389/fnagi.2023.1219987

**Published:** 2023-05-23

**Authors:** Constanza J. Cortes, John P. Thyfault, Heather M. Wilkins

**Affiliations:** ^1^Leonard Davis School of Gerontology, University of Southern California, Los Angeles, CA, United States; ^2^Department of Cell Biology and Physiology, KU Diabetes Institute and Department of Internal Medicine-Division of Endocrinology, University of Kansas Alzheimer's Disease Research Center, University of Kansas Medical Center, Kansas City, KS, United States; ^3^Kansas Center for Metabolism and Obesity Research, Kansas City, MO, United States; ^4^Center for Children's Healthy Lifestyles and Nutrition, Kansas City, MO, United States; ^5^Kansas City VA Medical Center, Kansas City, MO, United States; ^6^Department of Neurology, University of Kansas Alzheimer's Disease Center, University of Kansas Medical Center, Kansas City, KS, United States; ^7^Department of Biochemistry and Molecular Biology, University of Kansas Alzheimer's Disease Center, University of Kansas Medical Center, Kansas City, KS, United States

**Keywords:** Alzheimer's disease, systemic, cardiovascular, diabetes, fitness, liver, kidney

In the past decade, it has become increasingly evident that Alzheimer's disease (AD) risk can be modulated by multiple systemic factors. For example, diabetes, cardiovascular disease, and low cardiorespiratory fitness are associated with increased risk for AD, highlighting the previously undescribed role for whole body metabolism in AD pathogenesis (Kopf and Frolich, 2009; Baker et al., 2010; Morris et al., 2014, 2017, 2020; Vidoni et al., 2015; Gaitan et al., 2019; Nho et al., 2019; Tini et al., 2020). Growing evidence supports metabolic changes are observed within the brain and systemic tissues in AD, including from blood, skin, muscle, and adipose tissue (Parker et al., 1990; Parker, 1991; Kish et al., 1992; Curti et al., 1997; Bosetti et al., 2002; Cardoso et al., 2004; Arbones-Mainar et al., 2010; Mosconi et al., 2011; Fisar et al., 2016, 2019; Morris et al., 2021; Kim et al., 2022). Furthermore, recent work describes both liver and kidney function correlate with cognition and AD pathological biomarkers (Nho et al., 2019; Stocker et al., 2023). Overall, these findings highlight the role of systemic physiology in AD.

Our goal for this Research Topic was to highlight the role of systemic changes in AD. Although further studies are needed to understand if systemic changes are a cause or a consequence of AD, understanding systemic contributions to AD can provide novel targets to modify risk through lifestyle changes. Promoting studies which provide integral discussion on systemic physiology in AD is imperative to understand disease etiology and mitigate risk.

A seminal study in 2019 highlighted the association of elevated plasma liver enzyme biomarkers with poor cognition, increased AD risk, elevated brain Aβ burden, and reduced brain glucose metabolism (Nho et al., 2019). As a follow up to this study, in this issue Wu et al. examined the association of liver function with cognitive impairment using the Shenzhen aging-related disorder cohort. The study leveraged a cohort of 7,201 people over the age of 60 years old to examine cognition and blood liver enzyme biomarkers, alanine aminotransferase (ALT) and aspartate aminotransferase (AST). Wu et al. reported higher AST to ALT ratio in the cognitive impairment group compared to the non-impaired group. The cognitive impairment group also had significantly increased age, lower education level, and more female participants. Overall, this study highlights an association of reduced liver function with cognition, a new area of research for the field. Further studies are warranted to understand the role systemic liver physiology contributes to brain aging, cognition, and AD risk.

Renal function is associated with cognitive impairment, however the relationship between renal function and AD biomarkers is currently poorly understood. The cross-sectional study by Zhang et al. examined associations of estimated glomerular filtration rate (eGFR) with plasma biomarkers for AD. While plasma Aβ biomarkers did not associate with renal function measured by eGFR, plasma Tau and neurofilament light (NfL) biomarkers did. These data suggest that reduced renal function is associated with increased markers of neurodegeneration. Renal and hepatic function are directly associated with whole body metabolism, and these studies directly support a role for systemic metabolism in regulating brain aging.

The role of energy metabolism in AD is highly cited. Energy disruption is noted across multiple tissues including brain, muscle, skin, and blood. Multiple studies suggest that AD pathologies like Aβ and Tau can disrupt mitochondrial function and energy metabolism (Hansson Petersen et al., 2008; Swerdlow et al., 2014, 2017; Reddy and Oliver, 2019; Weidling et al., 2020; Wilkins et al., 2022; Zysk et al., 2023). Systemic mitochondrial dysfunction and disrupted energy metabolism are noted in AD subjects, even in tissues where AD pathologies are lacking. This suggests disrupted energy metabolism is upstream of AD pathologies, as proposed in the mitochondrial cascade hypothesis of AD (Swerdlow et al., 2014). Modulating energy metabolism and mitochondrial has become a major target for AD therapeutics. Pre-clinical studies and clinical studies support the principle that aerobic capacity and fitness can modulate energy metabolism.

Aerobic capacity and fitness (or the lack there-of) are risk factors for not only systemic diseases, like diabetes and cardiovascular disease, but also for cognitive decline and AD. To study the effects of intrinsic aerobic capacity, rat models were selectively bred for their capacity for voluntary wheel running (Kerr et al.). This breeding scheme yielded low voluntary wheel runner rats with low aerobic capacity and high voluntary wheel runner rats with high aerobic capacity. Kerr et al. examined cognition, hippocampal neurogenesis, and mitochondrial function in these rat models selectively bred for intrinsic aerobic capacity and physical activity. High aerobic capacity and physical activity benefited neurogenesis, hippocampal size, brain glucose metabolism, and brain mitochondrial respiration in female rats. These effects were not observed in male rats. Physical inactivity and aerobic capacity have heritable effects on brain health and aging, with increased susceptibility in females.

Thyroid function has been implicated in AD risk, especially in women. The thyroid is an essential organ that secretes hormones critical for energy metabolism homeostasis. Based on prior findings associating thyroid function with AD, Ma et al. described an association of dementia risk and cognitive impairment with thyroid disease using a meta-analysis systemic review (PROSPERO: CRD42021290105). Fifteen studies were included in the meta-analysis which suggested that hyperthyroidism and subclinical hyperthyroidism are associated with increased dementia risk, while hypothyroidism was not associated with dementia risk. This meta-analysis highlights a potential role of hyperthyroidism in dementia risk and the need to understand the relevance to disease pathogenesis.

Beyond changes in energy metabolism, systemic inflammation is also implicated in AD. Oral hygiene is an important factor in systemic inflammation. Furthermore, oral health is an important indicator for cardiorespiratory health and can directly impact risk for infections (Kotronia et al., 2021). Occlusal support is an indicator of dental health including tooth loss. Occlusal support allows mastication (chewing), food mixing with salivary and gastric enzymes facilitating digestion and nutrient absorption. Da et al. assessed cognitive function and occlusal support in community dwelling adults aged over 60 years using a cross-sectional study design. Individuals with poor occlusal support had an increased odds ratio for cognitive impairment when compared to those with good occlusal support. Data were adjusted for age, sex, education level, cigarette smoking, alcohol drinking, cardiovascular disease, and diabetes. Age mediated most of the association of occlusal support and cognitive impairment. Further studies are needed to understand the association of age, occlusal support, and cognitive impairment.

Collectively, the articles in this Research Topic emphasize the importance of understanding systemic contributions to AD (Figure 1). Understanding how systemic risk factors over lifespan can impact brain aging and cognition is critical to the field of AD. Systemic risk factors, such as diabetes, cardiovascular disease, and physical activity are modifiable and could greatly impact the impact of AD on the aging population.

**Figure 1:**
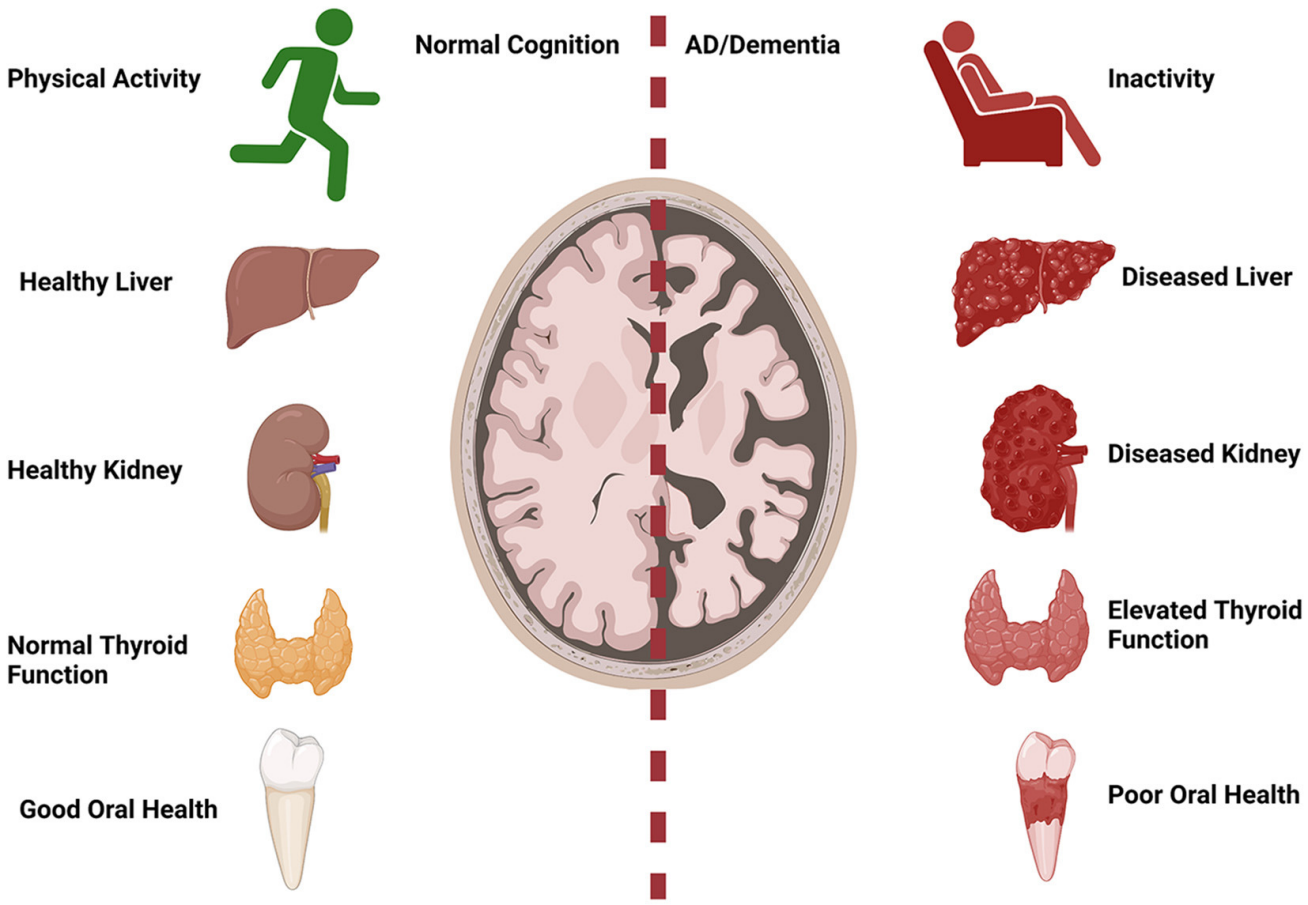
Systemic implications for AD. Normal cognition with aging is associated with increased physical activity, healthy liver and kidney function, normal thyroid function, and good oral health. AD/dementia risk is increased with physical inactivity, abnormal liver and kidney function, hyperthyroidism, and poor oral health. Created with BioRender.com.

## Author contributions

All authors listed have made a substantial, direct, and intellectual contribution to the work and approved it for publication.
